# Seminal fluid protein genes of the brown planthopper, *Nilaparvata lugens*

**DOI:** 10.1186/s12864-016-3013-7

**Published:** 2016-08-18

**Authors:** Bing Yu, Dan-Ting Li, Jia-Bao Lu, Wen-Xin Zhang, Chuan-Xi Zhang

**Affiliations:** State Key Laboratory of Rice Biology and Ministry of Agriculture Key Laboratory of Agricultural Entomology, Institute of Insect Science, Zhejiang University, Hangzhou, 310058 China

**Keywords:** *Nilaparvata lugens*, Seminal fluid protein, Proteome, UPLC/MS/MS

## Abstract

**Background:**

Seminal fluid proteins (SFPs) are produced mainly in the accessory gland of male insects and transferred to females during mating, in which they induce numerous physiological and post-mating behavioral changes. The brown plant hopper (BPH), *Nilaparvata lugens*, is an economically important hemipterous pest of rice. The behavior and physiology of the female of this species is significantly altered by mating. SFPs in hemipteran species are still unclear.

**Results:**

We applied high-throughput mass spectrometry proteomic analyses to characterize the SFP composition in *N. lugens*. We identified 94 putative secreted SFPs, and the expression levels of these proteins was determined from the male accessory gland digital gene expression database. The 94 predicted SFPs showed high expression in the male accessory gland. Comparing *N. lugens* and other insect SFPs, the apparent expansion of *N. lugens* seminal fluid trypsins and carboxylesterases was observed. The number of *N. lugens* seminal fluid trypsins (20) was at least twice that in other insects. We detected 6 seminal fluid carboxylesterases in *N. lugens* seminal fluid, while seminal fluid carboxylesterases were rarely detected in other insects. Otherwise, new insect SFPs, including mesencephalic astrocyte–derived neurotrophic factor, selenoprotein, EGF (epidermal growth factor) domain–containing proteins and a neuropeptide ion transport-like peptide were identified.

**Conclusion:**

This work represents the first characterization of putative SFPs in a hemipeteran species. Our results provide a foundation for future studies to investigate the functions of SFPs in *N. lugens* and are an important addition to the available data for comparative studies of SFPs in insects.

**Electronic supplementary material:**

The online version of this article (doi:10.1186/s12864-016-3013-7) contains supplementary material, which is available to authorized users.

## Background

Insect seminal fluid proteins (SFPs) are important for fertilization and are weapons for males in sexual competition, such as manipulating post-mating physiological and behavioral changes in females [1]. SFPs have complex structures and perform a diversity of functions [2]. Under natural selection and selection by females, and under male competition [2], the rapid evolution of SFPs has been observed [3]. The SFP compositions of insect species exhibit significant diversity, presumably enabling a variety of reproductive strategies. Week-long refractoriness toward further copulation and enhanced egg laying levels are generated by the seminal fluid sex peptide (Acp70A) pathway in *Drosophila melanogaster*, and sperm is required in this process [4–6]. An unknown mechanism leads to life-long behavioral changes in mosquitoes such as *Anopheles gambiae*, and sperm is not required [7]. The female behavior and physiology of multiple mating social insects are apparently unaffected by a single copulation, but SFPs may respond to the long-term storage of sperm and sperm competition after copulation [8, 9].

The brown planthopper (BPH), *Nilaparvata lugens* Stål (Hemiptera: Delphacidae), is one of the most serious insect pests of rice in Asia [10]. Asian countries have continually experienced serious outbreaks of BPH although new BPH-resistant rices, new insecticides, as well as integrated pest management programs are used. Mated BPH females display stimulated egg laying levels [11] and almost life-long refractoriness to further insemination [12]. The sex peptide model, as used to describe post-mating behavior in *D. melanogaster*, may not provide a reasonable explanation for post-mating behavior in *N. lugens*. At present, chemical control remains the first choice for *N. lugens* management [13]. Seminal fluid might play a part in the rapid establishment of drug resistance. Insecticide (triazophos and deltamethrin)-treated male *N. lugens* had higher protein content than untreated males; treated males also transferred more SFPs to mated females [14, 15].

Proteomic approaches to elucidating the function of SFPs have been carried out on several insect species, including *Apis mellifera* [8], *D. melanogaster*, *D. simulans*, *D. yakuba* [16], *Tribolium castaneum* [17], *Aedes aegypti* [18], *Aedes albopictus* [19], *Teleogryllus oceanicus* [20], *Heliconius erato*, and *H. melpomene* [21]. Proteomic research on SFPs has not been performed for any hemipterous species to date, such as *N. lugens*. Furthermore, the functions of insect SFPs have been poorly studied in insects other than *D. melanogaster*, despite major differences in reproductive physiology exist between species. More seminal protein information from multiple insects could provide more insights into the evolutionary patterns of reproductive traits [22]. As more is learned about the reproductive biology of specific arthropods, their SFPs may provide tools or targets for the control of disease vectors and agricultural pests [22]. The *N. lugens* seminal fluid proteome could benefit research into the reproductive physiology of *N. lugens* that uses tools such as RNA interference (RNAi). Illustrating the molecular interactions between SFPs and *N. lugens* females may aid researchers in identifying molecular targets for pest control, as the regulation of female behaviors after mating appears to be long-lasting in *N. lugens*.

**Fig. 1:**
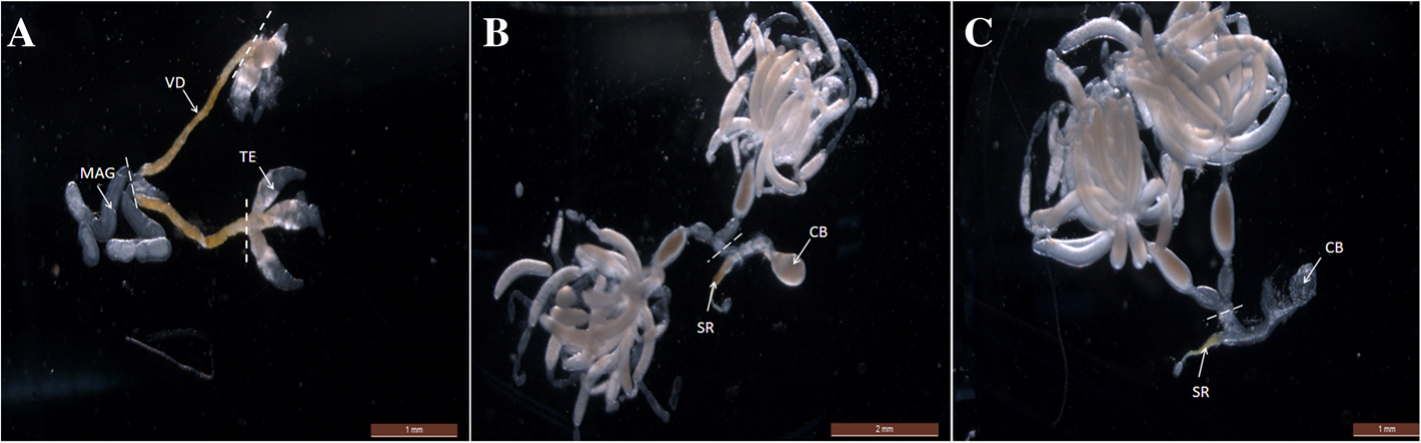
Tissues dissected for sample preparation. **a** Male reproductive tract (MRT). The whole MRT and dissections of MRT (including TE, VD, and MAGs) dissected from 40 males were collected for RT-qPCR. MAGs dissected from 50 males were collected for MAG protein sample preparation. Each tissue was dissected from the dotted line. **b** Mated female reproductive tracts. Copulatory bursas (CB) and seminal receptacles (SR) dissected from 50 mated females were collected for mated-FRT protein sample preparation. Each tissue was dissected from the dotted line. **c** Unmated FRTs. The whole FRT dissected from 40 unmated females were collected for RT-qPCR. Copulatory bursas (CB) and seminal receptacles (SR) dissected from 50 females were collected for unmated-FRT protein sample preparation. Each tissue was dissected from the dotted line

Recently, the whole genome sequences and gene annotation information for *N. lugens* were described [10]. Gene expression information regarding developmental stages, wing dimorphism, sex differences, and tissues was collected using next-generation high-throughput Illumina technology [13, 23–26]. The male reproductive tract (MRT) of *N. lugens* comprises two testes (TE), two vas deferens (VD), two male accessory glands (MAGs), and one ejaculatory duct (Fig. 1). Sperm are produced by the TE, and SFPs are produced primarily by the MAGs. In this study, transcriptomic analysis of the *N. lugens* MRT was performed, and gene expression information concerning the MAG was obtained using a tag-based digital gene expression (DGE) system. We used UPLC/MS/MS to identify the transferred SFPs of *N. lugens*.

## Methods

### Insects

The *N. lugens* strain was originally collected from a rice field located in the Huajiachi Campus of Zhejiang University in Hangzhou, China. The insects used in this experiment were the offspring of a single female. Insects were reared on rice seedlings at 28 °C (Xiu shui 128) under a 12:12 h light: dark photoperiod.

### Preparation of *N. lugens* MRT transcriptome database

*N. lugens* males were anesthetized on ice for 20 min and dissected under a Leica S8AP0 stereomicroscope. The whole MRT (including the TE, VD, and MAGs) (Fig. 1) were isolated and quickly washed in a diethylpyrocarbonate (DEPC)-treated phosphate-buffered saline (PBS) solution (137 mM NaCl, 2.68 mM KCl, 8.1 mM Na_2_HPO_4_, and 1.47 mM KH_2_PO_4_ at pH 7.4) and were immediately frozen at −80 °C. The MRT sample was used for transcriptome and DGE sequencing, and the MAG sample was used for DGE sequencing.

Total RNA was isolated from *N. lugens* MRT and MAG using TRIzol reagent (Invitrogen, Carlsbad, CA, USA) following the manufacturer’s instructions. Sequencing and assembly of transcriptome reads, including DGE library reads, was performed using Illumina HiSeq™2000 and Trinity (v2012-10-05), respectively, and the annotation of unigenes were performed as described previously [23]. The longest assembled transcripts of each gene were taken as unigenes. The readcount of each unigenes was normalized to RPKM (Reads Per Kilo bases per Million mapped Reads) to display the expression level of each unigene. The coding sequence (CDS) of each unigene was analyzed using blastx and estscan (3.03). The generated peptide database was used to support the proteomic analysis.

### Seminal fluid protein sample preparation

Seminal fluid samples were collected from males and mated females, and soluble protein samples were collected from unmated females (all individuals were 4–7 days post-eclosion). Mated females were obtained by placing one female in a glass tube containing a rice seedling with one male for 2 h. The female copulatory bursa (CB) and seminal receptacle (SR) (Fig. 1) were dissected in PBS solution and squeezed using grinding rod in 100 μl PBS with 1 % protease inhibitor cocktail (Thermo, USA). Reproductive tracts from ≈ 50 females were pooled for each biological replicate. MAGs (Fig. 1) were dissected using the same method, and MAGs from ≈ 50 males were collected for each biological replicate. Samples were centrifuged at 12,000 rpm for 20 min at 4 °C. The supernatant was transferred to a separate tube and stored at −80 °C. Three replicates were prepared for each kind of sample including the MAG, the mated-female reproductive tract (FRT) and the unmated-FRT.

A filter aided sample preparation (FASP) method was used for the preparation of samples [27]. Samples were added to 3 kD ultrafiltration centrifuge tubes (Millipore), and centrifuged at 14000 g for 20 min. 100 μl of UA solution [8 M Urea (Sigma), 0.1 M Tris/HCl pH 8.5, 1 % EDTA (Thermo), 1 % protease inhibitor Cocktail (Thermo)], and centrifuged at 14000 g for 20 min; this step was repeated twice. 2 μl DTT (Sigma) (200 mM) was added. Samples were vortexed for 1 min and incubated at 37 °C for 1 h. 20 μl iodoacetamide (Sigma) (200 mM) was then added. Samples were vortexed for 1 min and incubated at 25 °C for 1 h in the dark. Samples were centrifuged at 14000 g for 20 min. To each sample, 100 μl UA was added. Samples were then centrifuge at 14000 g for 20 min; this step was repeated once. 200 μl NH_4_HCO_3_ (Sigma) (0.05 M) was added, and samples were centrifuged at 14000 g for 20 min; this step was repeated twice. The remaining sample was moved into a 10 kD ultrafiltration centrifuge tube, and 40 μl NH_4_HCO_3_ (0.05 M) and trypsin (Promega) (5 μg in total) were added. Samples were incubated at 30 °C for 12 h, and then centrifuged at 14000 g for 20 min. 40 μl NH_4_HCO_3_ (0.05 M) was added; then samples were centrifuged at 14000 g for 30 min. Filtered liquid was removed into a 1.5 ml centrifuge tube and dehydrated in a vacuum freeze-drying device. Dehydrated samples were dissolved in 25 μl 0.1 % formic acid (Sigma). The concentrations of the dissolved peptide solutions were analyzed by A280 absorption using a NanoDrop UV–vis spectrophotometer (Thermo Fisher Scientific, Waltham, Massachusetts, USA).

### UPLC/MS/MS methods and data analyses

The peptide mixtures were injected onto the trap column at a flow rate of 10 μl/min for 2 min (2 μg) using a Thermo Scientific Easy nanoLC 1000. The trap was equilibrated at a maximum pressure of 500 bar for 12 μl, followed by column equilibration at a maximum of 500 bar for 3 μl before beginning the gradient elution of the column. The samples were subsequently eluted using the following five-step linear gradient (A: ddH2O with 0.1 % formic acid,B: ACN with 0.1 % formic acid): 0–10 min, 3–8 % B; 10–120 min, 8–20 % B; 120–137 min, 20–30 % B; 137–143 min, 30–90 % B; and 143–150 min, 90 % B. The column flow was maintained at 250 nL/min. The chromatographic system was composed of a trapping column (75 μm × 2 cm, nanoviper, C18, 3 μM, 100 Å) and an analytical column (50 μm × 15 cm, nanoviper, C18, 2 μM, 100 Å). Data collection was performed using a Thermo LTQ-Orbitrap Velos Pro equipped with a Nanospray Flex ionization source and a FTMS (Fourier transform ion cyclotron resonance mass spectrometry) analyzer combined with a Thermo LTQ-Orbitrap Elite equipped with an ion trap analyzer. The parameters for FTMS were as follows: Data collection at 60 K for the full MS scan, positive polarity, data type profile, and then proceeded to isolate the top 20 ions for MS/MS by CID (1.0 m/z isolation width, 35 % collision energy, 0.25 activation Q, 10 ms activation time). The scan range was set as 300 m/z first mass and 2000 m/z last mass. The parameters for the ion trap analyzer were the normal mass range, rapid scan rate, and centroid data type.

A SEQUEST HT search engine configured with a Proteome Discoverer 1.4 workflow (Thermo Fischer Scientific, Bremen, Germany) was used for mass spectrometer data analyses. An *N. lugens* MRT peptide database generated from transcriptome unigene sequences database containing 17902 sequences were configured with SEQUEST HT for dataset searches. The search parameters included 10 ppm and 0.8 Da mass tolerances for MS and MS/MS, respectively, trypsin as the proteolytic enzyme with two allowed missed cleavages, oxidation and deamidated as dynamic modifications, and carbamidomethyl as a static modification. Furthermore, the peptides were extracted using high peptide confidence. 1 % FDR (False discovery rate) was calculated using a decoy database by searching both the MRT peptide sequence and the decoy database.

### Identification of seminal fluid proteins of *N. lugens*

High confidence proteins were identified with the following standards: 1) Proteins identified from more than two samples (proteins derived from at least two MAG samples, two unmated-FRT samples and two mated-FRT samples) were predicted to be “true” detected proteins. 2) Seminal fluid proteins must have been identified from both MAG and mated-FRT samples. 3) We tested for predicted secretion signal sequences of detected proteins using SignalP 4.1 (www.cbs.dtu.dk/services/SignalP/). Some sequences had a “bad” coding sequence CDS prediction (Lost in N-terminal), in which the signal peptide was not be predicted from the sequence. We re-predicted CDS sequences for proteins with no signal peptide using ESTScan (http://myhits.isb-sib.ch/cgi-bin/estscan) from unigene nucleotide sequences, and performed Signalp detection with new predicted CDS sequences for improved signal peptide detection. Proteins possessing a signal peptide were considere d to be secreted proteins. 4) Some proteins did not possess a signal peptide. Proteins without signal peptides that were not detected in unmated-FRT samples and showed male-specific expression (an analysis of the male-specific expression of unigenes was performed as described previously [13]) were also predicted to be secreted. 5) In addition, other proteins that were not predicted to be secreted and had homologues in the SFPs of other insects were classied as unconfirmed SFPs.

### Annotation of seminal fluid proteins and comparison with other insects

In addition to machine annotation, we performed a manual annotation for the sequences detected. Blast results from NCBI, conserved domains, and GO terms were used in combination to annotate proteins. Brief descriptions from NCBI, SMART (http://smart.embl-heidelberg.de/) descriptions of conserved domains, and functional descriptions of gene names from UniProtKB (http://www.uniprot.org/help/uniprotkb) were used to classify the functions of each sequence. Based on these matches, proteins were classified into one of the following categories: cell structure (including cell structure proteins and their binding proteins), metabolism, protein modification machinery, proteolysis regulators (proteases and protease inhibitors), signal transduction (including hormones), transporters and protein export machinery, and RNA and protein synthesis (transcription factors, transcription machinery, and protein synthesis enzymes). Proteins that were classified into different categories were classified as “other” (including salivary proteins, chitin binding proteins, binding proteins, proteasome machinery, protein kinases, ubiquitination pathway proteins, protein phosphatases, and oxidoreductases). Proteins that were not assigned a function were classified as “unknown”.

Seminal fluid proteome sequences of *D. melanogaster*, *A. aegypti*, *A. albopictus*, *A. mellifera* and *Homo sapien* [28] were chosen for comparison with SFPs of *N. lugens*. SFP sequences of *D. melanogaster* were extracted from Flybase (http://flybase.org/) using IDs given from reference [16]. SFPs sequences of *A. aegypti* were extracted from Ensembl Metazoa (http://metazoa.ensembl.org/info/website/ftp/index.html) using IDs given from reference [18]. SFP sequences of *A. albopictus* were directly given by reference [19]. SFPs sequences of *A. mellifera* were extracted from NCBI (http://www.ncbi.nlm.nih.gov/sites/batchentrez) using IDs given from reference [8]. Signal peptides of these proteins were identified as mentioned in 2.5. And prediction of conserved domains of predicted protein domains was using the Batch Web CD-Search Tool (http://www.ncbi.nlm.nih.gov/Structure/bwrpsb/bwrpsb.cgi). *N. lugens* SFPs possessing the same conserved domain with other insect SFPs were marked as “Domain”. The rest proteins with blastp (Evalue < 10^−5^) hits with other insect SFPs were marked as “Blast”. The same method was used for comparison of SFPs between insect species.

To locate the detected proteins in the *N. lugens* genome scaffold sequences, we run a megablast with Evalue < 10^−20^, and indentity > 95 % between detected proteins and scaffold sequences.

### Phylogenetic analysis

The functional serine protease domains of the *N. lugens* seminal fluid trypsins were aligned with seminal fluid trypsins of other insect species using the ClustalX program. The phylogenetic tree was constructed by the maximum likelihood (ML) method using the program Mega 5.05 (http://www.megasoftware.net/). Homologous relationships were determined using bootstrap analysis with 1000 replications.

### Reverse-transcription quantitative PCR (RT-qPCR) analysis

MRT, FRT, and dissections of MRT (including testes, vas deferens, and male accessory glands) (Fig. 1) were dissected from males (4–7 days post-eclosion). As the mRNA quantity of an individual tissue is extremely low, tissues dissected from 40 individuals were pooled into each tissue sample, respectively. RT-qPCR was performed according to the method of [29]. Primers used in RT-qPCR for the tissue specific expressions of seminal fluid protein genes are given in Additional file 1: Table S1.

## Results

### MRT transcriptome sequencing and assembly

Illumina sequencing produced 2.59 GB nucleotides. The quality of this transcriptome sequence was high, with a Q20 percentage (the percentage of sequences with a sequencing error rate of 0.03 %) and GC content of 98.21 % and 40.08 %, respectively. These short reads were assembled into 57568 transcripts with a mean length of 741 bp. Ultimately, we obtained 37443 unigenes with a mean size of 641 bp and lengths ranging from 201 to 9670 bp. Annotation of these sequences revealed that 13089 (35 %) sequences were annotated in the NR database, 10156 (27 %) sequences were annotated in the SwissProt database, 10589 (28 %) sequences were annotated in the GO database, and 8301 (22 %) sequences were annotated in the KOG database. Of these, 523 sequences showed male-specific expression. A peptide database with 17902 sequences was generated as a query database for the raw data of the proteome (Table 1).

**Table 1:** Summary of transcriptome sequence datasets of the BPH reproductive tract

Group	Number of unigenes
Total unigene	37443
MRT expression	33798
MAG expression	19772
Male specific expressed	523
Obtained peptide database	17902

The longest assembled transcripts of each gene were taken as unigenes. Only unigenes with RPKM value larger than 0.3 were counted as expressed. Owing to the accomplishment of the transcriptomic sequencing of the differences between the *N. lugens* development and sex genes in our previous study [11], we are able to use the predicted SFP genes as reference sequences to map the transcriptomic datasets and to analyze the expression sex-specific genes. The coding sequence (CDS) of each unigene was analyzed using blastx and estscan (3.03). The generated peptide database was used in proteome query

Two DGE libraries from the MRT and MAG were also sequenced, generating approximately 0.64 GB clean tags for each library. Among the clean tags, approximately 92 % of sequences could be mapped to unigenes in each library. A total of 33798 unigenes expressed in the MRT had a RPKM value > 0.3; 19772 unigenes expressed in the MAG had a RPKM value > 0.3 (Table 1). The MRT transcriptome yielded a peptide database used in proteome sequencing, and the MRT and MAG DGE databases provided the expression levels of detected protein-coding genes in the MRT and MAG.

### Proteins transferred to females during mating

We identified a total of 218 putative SFPs from both the MAG and mated-female reproductive tract (FRT) samples. Of these, 65 sequences were not detected in unmated-FRT samples and showed male-specific expression. Fifty-five of the 65 sequences had a signal peptide; the remaining 10 proteins showed high expression in the MAG. In addition, 29 proteins that were detected in both mated- and unmated-FRT samples also had a signal peptide. These proteins were predicted to be secreted in the MAG. Eventually, a total of 94 proteins were predicted secreted SFPs (Table 2).

**Table 2:** Identified secreted SFPs of *N. lugens*

Genes	Function category	*D. melanogaster*	*A. aegypti*	*A. albopictus*	*A. mellifera*
Cell structure					
Annexin	Cell structure		T	T	
Lipid related					
Prosaposin	Metabolism: lipid			T	
Carboxylesterase (7)	Metabolism: lipid	T			
Lysosomal acid lipase	Metabolism: lipid	T			
Pancreatic lipase-related protein 2-like	Metabolism: lipid	T	T	T	
Apolipoprotein D (2)	Transporters and protein export machinery	T			
Apolipophorin-III	Transporters and protein export machinery and antibacterial				
Metabolism					
beta-hexosaminidase		T			
Chitinase (2)	Metabolism: carbohydrate				T
Alpha mannosidase	Metabolism: carbohydrate		T	T	T
Soluble trehalase	Metabolism: carbohydrate				
Nucleoside diphosphate kinase	Metabolism: nucleotide		T	T	
Carbonic anhydrase	Metabolism: others				
Protein modification machinery					
Heat shock 70 kDa protein (2)	Protein modification machinery		T	T	T
FKBP-type peptidyl-prolyl cis-trans isomerase (2)	Protein modification machinery	T	T		
Gamma-interferon-inducible lysosomal thiol reductase (2)	Protein modification machinery			T	T
Protein disulfide isomerase (3)	Protein modification machinery	T		T	
Calreticulin	Protein modification machinery			T	
Mesencephalic astrocyte-derived neurotrophic factor	Protein modification machinery				
Endoplasmin	Protein modification machinery			T	T
Proteolysis regulators					
Furin-like protease	Proteolysis regulators: protease		T	T	
Aminopeptidase	Proteolysis regulators: protease	T	T	T	T
Zinc carboxypeptidase	Proteolysis regulators: protease				
glutamate carboxypeptidase	Proteolysis regulators: protease				
angiotensin-converting enzyme	Proteolysis regulators: protease		T	T	T
Lysosomal Pro-X carboxypeptidase	Proteolysis regulators: protease				
Glutaminyl-peptide cyclotransferase-like	Proteolysis regulators: protease			T	
Aspartyl protease (2)	Proteolysis regulators: protease			T	
Cathepsin B	Proteolysis regulators: protease	T		T	
Serine protease	Proteolysis regulators: protease				
Trypsin (20)	Proteolysis regulators: protease	T	T	T	T
SERPIN (2)	Proteolysis regulators: protease inhibitor	T	T	T	
Kazal type serine protease inhibitors(smart)	Proteolysis regulators: protease inhibitor				T
Cathepsin propeptide inhibitor(smart)	Proteolysis regulators: protease inhibitor	T		T	
Pacifastin inhibitor(smart)	Proteolysis regulators: protease inhibitor				
Carboxypeptidase inhibitor precursor(blast)	Proteolysis regulators: protease inhibitor	T			
Signal transduction					
Chemosensory protein (2)	Signal transduction	T			T
Phosphatidylethanolamine binding protein	Signal transduction	T	T		T
Renin receptor	Signal transduction				T
Ion transport peptide	Signal transduction				
Other					
Cysteine-rich secretory protein (2)	Other: salivary	T	T	T	T
Unknown	other: oxidoreductase				
Protein with chitin binding Peritrophin-A domain (4)	Other: chitin binding	T			
Dumpy(blast) (3)	Other: binding				
Selenoprotein	Other: sperm quality				
Unknown					
Hypothetical protein(blast) (3)	Unknown		T	T	
Unknown (5)	Unknown				

Numbers after the gene names stand for the number of proteins detected. Conserved domains of insect SFPs were identifed using the Batch Web CD-Search Tool (http://www.ncbi.nlm.nih.gov/Structure/bwrpsb/bwrpsb.cgi). A local blastp analyse (evalue = 1e-5) were performed between BPH SFPs with other insect SFPs. “T” stands for the sequences possess the same conserved domain or show blastp (1e-5) results with SFPs with other insect SFPs. D. *melanogaster*, *Drosophila melanogaster*; *A. aegypti*, *Aedes aegypti*; *A. albopictus*, *Aedes. albopictus*; *A. mellifera*, *Apis mellifera*

One hundred and twenty-four of the 218 proteins had identical conserved domains or were aligned in blastp results with SFPs detected from other insects; this latter class of proteins contained no signal peptide and did not show male-specific expression (Additional file 2: Table S2). Homologues of these proteins had been detected in the SFPs of *D. melanogaster*, *A. aegypti*, *A. albopictus*, or *Apis mellifera* previously and were classified as unconfirmed SFPs. Whether these proteins are “true” transmitted seminal proteins in *N. lugens* could not be confirmed due to technical limitations (transmitted SFPs could not be distinguished from FRT proteins).

**Fig. 2:**
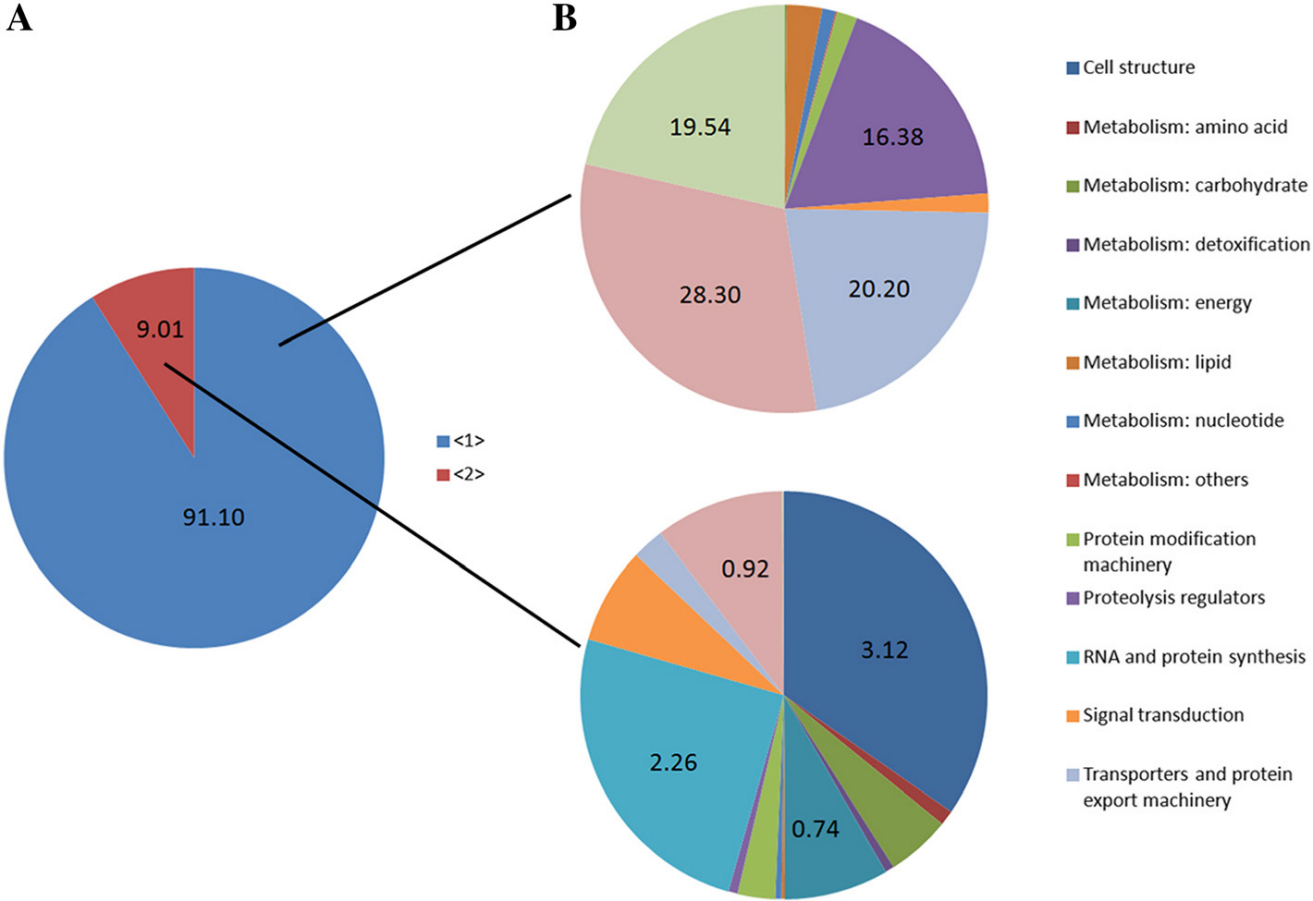
Functional categories of detected proteins from the MRT and FRT of *N. lugens*. **a** The percentage of protein expression level in the MAG associated with each group. (The accumulated RPKM value of unigenes in each group/the accumulated RPKM value of the whole unigene). <1 > Blue columns, proteins predicted to be secreted SFPs. <2 > Red columns, proteins not predicted to be secreted SFPs but that have the same conserved domain or show blastp (1e-5) results with SFPs of *D. melanogaster*, *A. aegypti*, *A. albopictus*, and *Apis mellifera* seminal fluid. **b** The percentage of protein expression level in the MAG associated with each functional category. (The accumulated RPKM value of unigenes in a functional group/the accumulated RPKM value of the whole unigene)

Through sequence annotation, the 218 proteins were classified into different functional groups. By combining protein annotation and the expression patterns of these proteins in the MAG and MRT, we found that predicted secreted SFPs and unconfirmed SFPs exhibited large differences in expression patterns and functional group classifications. MAGs are the main production center for SFPs. The proteins predicted to be secreted showed high expression in the MAG; predicted secreted proteins accounted for 91.10 % of the accumulated RPKM value of the 218 genes. Some proteases, two apolipoprotein D proteins, two cysteine-rich secretory proteins, two peritrophin A–type chitin-binding proteins, three dumpy proteins, a nucleoside diphosphate kinase, a chemosensory protein, and four proteins with unknown function showed extremely high expression in the MAG (RPKM value > 1000). When analyzing the numbers of genes in each functional group, we found that proteolysis regulators represented the largest percentage of secreted proteins (37, 16.97 %); this was followed by protein modification machinery (12, 5.50 %), proteins with other functions (10, 4.59 %), and lipid metabolism proteins (10, 4.59 %) (Additional file 3: Table S3 and Fig. 2).

Most of the unconfirmed SFPs showed very low expression levels in the MAG; these genes accounted for only 9.01 % of the accumulated RPKM value of the 218 genes. Only several genes encoding cell structure proteins (such as actin, tubulin, myosin, profilin, and tropomyosin), proteins involved in RNA and protein synthesis (such as ribosomal proteins and translation initiation factors), energy metabolism proteins (such as cytochrome c, ATP synthase, and malate dehydrogenase), and other proteins (two thioredoxin genes in particular) (Fig. 2 and Additional file 3: Table S3) had relatively higher expression levels in the MAG. Most of these proteins are ubiquitous within tissues and throughout the developmental stages. We could not distinguish these proteins from FRT proteins.

One hundred and ninety-six of the 218 proteins had homologues in the SFPs in four other insect species; 73, 91, 114, and 77 homologous proteins were identified in *D. melanogaster*, *A. aegypti*, *A. albopictus*, and *Apis mellifera*, respectively. Among the 196 *N. lugens* proteins, only trypsins, cysteine-rich secretory proteins, a low-density lipoprotein receptor, and an aminopeptidase were found in all insect species. In *N. lugens*, the low-density lipoprotein receptor and the aminopeptidase were not predicted to be secreted; these two proteins also showed very low expression levels in the MAG.

**Fig. 3:**
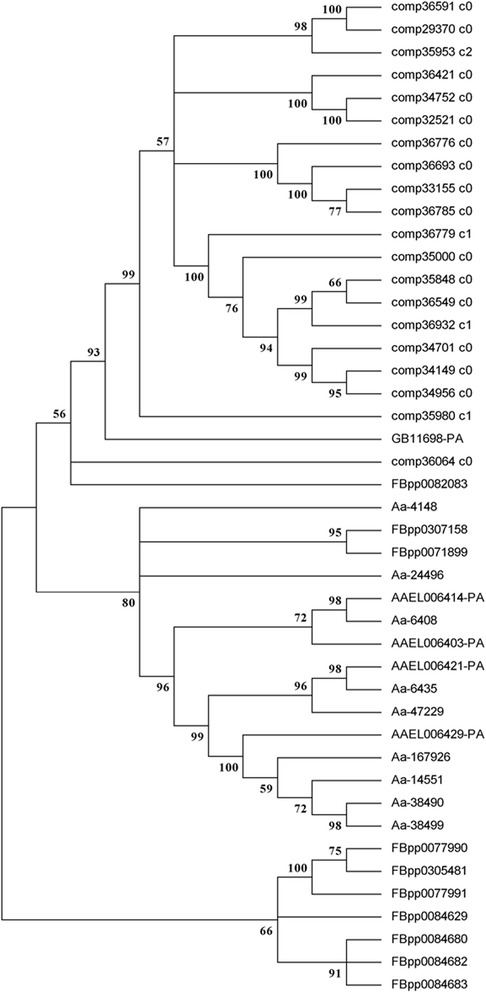
Phylogenetic analysis of insect seminal fluid trypsins. The phylogenetic tree was constructed based on the deduced amino acid sequences of the conserved domains of seminal fluid trypsin genes by maximum likelihood using Mega 5.05 (http://www.megasoftware.net/). The Jones-Taylor-Thornton (JTT) model for amino acid substitution was used, while a test of phylogeny was carried out using bootstrap analysis with 1000 replications. Sequences starting with “comp” stand for transcriptome unigene IDs of seminal fluid trypsins in *N. lugens*. Sequences starting with “FBpp” stand for *D. melanogaster* genome peptide IDs of seminal fluid trypsins (http://flybase.org/). Sequences starting with “AAEL” stand for *A. aegypti* genome peptide IDs of seminal fluid trypsins (http://www.vectorbase.org/). Sequences starting with “GB” stand for *Apis mellifera* genome peptide IDs of seminal fluid trypsins (http://www.ncbi.nlm.nih.gov/). Sequences starting with “Aa” stand for *A. albopictus* seminal fluid trypsins IDs

A much larger number of trypsins (20) were detected in *N. lugens* seminal fluid than in other insects (*D. melanogaster*, 10; *A. aegypti*, 4; *A. albopictus*, 11; *Apis mellifera*, 1) (see the phylogenetic tree in Fig. 3). As the phylogenetic tree shows, all *N. lugens* seminal fluid trypsins were on the same branch. Although the SFP genes evolve rapidly, one *D. melanogaster* trypsin showed a closer relationship with *N. lugens* seminal fluid trypsins, and two *D. melanogaster* trypsin showed a closer relationship with mosquito seminal fluid trypsins. The seminal fluid trypsins from the *A. aegypti* and *A. albopictus* were all mixed together. The duplication of *N. lugens* seminal fluid trypsins (including other *N. lugens* SFPs) in the same scaffold was observed (Additional file 2: Table S2). Furthermore, the number of carboxylesterases was also expanded in *N. lugens*. Only one carboxylesterase was identified in the *D. melanogaster* SFPs, and carboxylesterase was not detected in other insects.

Seven proteins detected in *N. lugens* had not been previously detected in other insect SFPs. These included lysosomal Pro-X carboxypeptidase, carboxypeptidase E, carboxypeptidase Q, prolylcarboxypeptidase, mesencephalic astrocyte–derived neurotrophic factor (MANF), selenoprotein, EGF (epidermal growth factor)-domain containing proteins, and an ion transport peptide–like (ITPL) peptide.

Considering SFPs may undergo enzymatic digestion or other alternations in the female reproductive system, thus, they may not be detected from female samples. Accordingly, we analyzed the signal peptide of the proteins that were only detected from MAG samples. We identified 14 proteins with signal peptides, and their information, including their sequences, is provided in Additional file 4: Table S8.

### Expression profile analysis of SFP genes by RT-qPCR

We tested the expression profiles of 34 genes in the MRT, FRT, and dissections of the MRT using RT-qPCR. Thirty-one genes showed MRT-specific or -biased expression (Additional file 5: Figure S1). Of these 31 genes, we have previously reported that 9 trypsins are specifically expressed in the MRT [29] (not shown in figure). When analyzing the expression of these sequences within the MRT, most of the MRT-specific or -biased genes also showed MAG-specific or -biased expression. Two exceptions were apolipophorin III, which showed the greatest expression in the VD, and a carboxylesterase, which showed high expression in both the VD and the MAG (Additional file 5: Figure S1). The qPCR results were consistent with the gene expression level results of the transcriptome.

## Discussion

The presence of a signal peptide is the classic method of predicting whether a protein is secreted. In *D. melanogaster*, 142 SFPs were detected; of these, 112 had a signal peptide. Seminal fluid proteome studies in other insects have reported a much lower proportion of proteins with signal peptides (29 of 93 detected proteins in *A. aegypti*, 57 of 198 detected proteins in *A. albopictus*, 20 of 53 detected proteins in *Apis mellifera*). These intracellular or membrane-bound proteins are predicted to be secreted via “apocrine secretion”; cells in the posterior portion of the glands are thought to secrete proteins through granules and/or via the rupture of the cell membrane [18], though some unsolved problems exist concerning “apocrine secretion”: 1. The biological importance of these proteins in the female reproductive tract remains to be demonstrated; 2. Proteins may be randomly included in seminal fluid during “apocrine secretion”; 3. The inclusion in the database of peptide sequences lacking an N-terminus may have complicated the prediction of signal peptides. The high proportion of proteins with a signal peptide in the *D. melanogaster* SFP proteome may have been due to the high quality of the *D. melanogaster* protein database. In this study, we combined the proteome and expression levels of detected proteins in the MAG and found that proteins with more reliable evidence were secreted (84 proteins with a signal peptide and 10 proteins not detected in unmated female MRT) and showed much higher expression in the MAG than other proteins did. The high expression of these proteins in the MAG is in accordance with the theoretical SFP expression profile.

In *D. melanogaster*, four trypsins are required in the sex peptide pathway, and C-type lectins are also needed in this pathway. In *N. lugens*, although a large number of trypsins was detected, we did not find C-type lectin in *N. lugens* seminal fluid. Otherwise, astacin family zinc metalloprotease [30], a protein required in fly ovulin (a prohormone-like SFP stimulating ovulation) [31], was not detected in *N. lugens* seminal fluid. In addition, we performed a tblastn using *D. melanogaster* ovulin and sex peptide against *N. lugens* MRT transcriptome unigene sequences, genome coding sequences, and genome DNA sequences, but no homologous sequences were identified. This indicates that the typical sex peptide and ovulin pathway are may not be present in *N. lugens*. The molecular mechanisms behind the post-mating phenomena of *D. melanogaster* and *N. lugens* may differ.

An angiotensin-converting enzyme was detected in *N. lugens*: this SFP is critical for increasing female egg laying in *T. castaneum* [17] and is related to female fecundity in *Anopheles stephensi* [32]. Two additional proteins associated with angiotensin were also detected in *N. lugens* seminal fluid. One is a renin receptor (detected in *N. lugens* and *Apis mellifera*) that induces the conversion of angiotensinogen to angiotensin I [33, 34]. The second protein is a lysosomal Pro-X carboxypeptidase (only detected in *N. lugens*) that can cleave C-terminal amino acids linked to proline in peptides such as angiotensin II in response to inactivation [35, 36].

Some new insect SFPs were identified in this proteome research, including new seminal fluid proteases, selenoprotein, secreted proteins containing EGF domains, a secreted neurotrophic factor MANF, and a neuropeptide ITPL protein. Selenoproteins were only recently detected in human seminal fluid; they are likely important for protecting sperm during storage [37]. In blastp analysis, secreted proteins containing EGF domains detected in *N. lugens* seminal fluid aligned with dumpy from *Drosophila*. Dumpy is a huge protein with an EGF domain repeat predicted to be a membrane-anchored fiber almost a micrometer in length; the EGF domain is involved in cell interactions [38]. A secreted protein with an EGF domain was found to bind to the surface of sperm and was important in sperm–egg binding in mice [39]. MANF protects and repairs the dopaminergic neurons. It is up-regulated in response to misfolded proteins, and it protects against various forms of endoplasmic reticulum stress in non-neuronal cells [40].

**Fig. 4:**
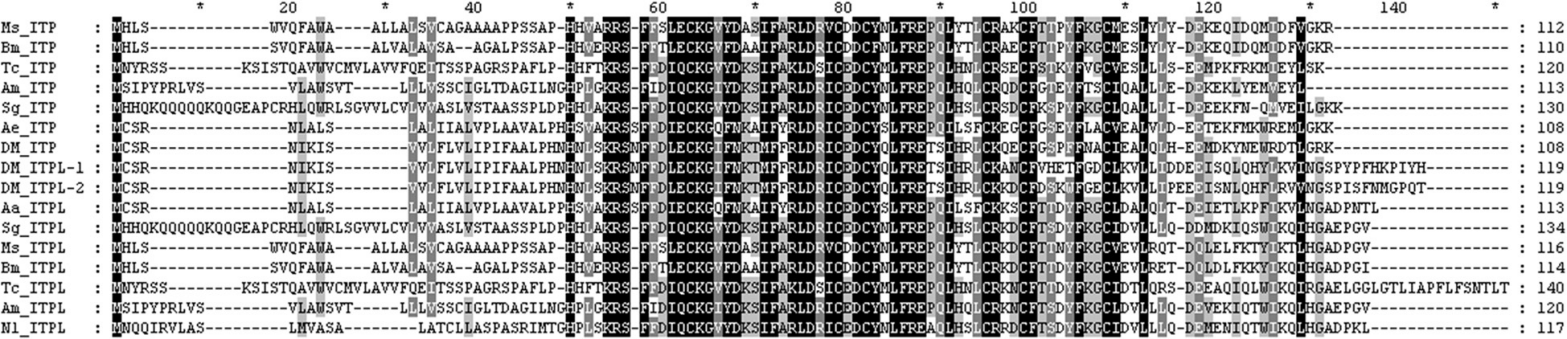
Alignment of NLITPL aa sequence with homologous ITP/ITPL other species. The *N. lugens* ITP (L) amino acid sequence was aligned with ITP (L) of other species using the ClustalX program and displayed using GeneDoc program. The sequences used in the alignment were as follows (species, GenBank accession number): Ms-ITP/ITPL (*Manduca sexta*, AAY29657.1, AAY29658.1), Bm-ITP/ITPL (*Bombyx mori*, NP_001106139.1, XP_012544167.1), Sg-ITP/ITPL (*S. gregaria*, Q26491.1, Q26492.1), Am-ITP/ITPL (*Apis mellifera*, XP_006571870.1, XP_006571871.1), Aa-ITPL (*A. aegypti*, XP_001653959.1), DM-ITP/ITPL-1/ITPL-2 (*D. melanogaster*, NP_001163293.1, NP_001036569.2, NP_611931.3), Tc-ITP/ITPL (*T. castaneum*, XP_008195073.1, XP_008195066.1), Ae-ITP (*Acromyrmex echinatior*, XP_011069312.1)

Most interesting is the discovery of the new seminal fluid neuropeptide, ITPL. IPTs were originally identified in *Schistocerca gregaria* and are regulators of ion and fluid transport across the ileum. However, ITPL lacks this activity due to C-terminal disparity (Fig. 4). ITP/ITPLs are highly conserved neuropeptides in insects and crustaceans and are grouped into the crustacean hyperglycemic hormones (CHH) [41, 42]. Several insect studies have suggested that ITP functions in ecdysis in *Manduca sexta* [43] and in *D. melanogaster* clock neurons [44]. RNAi of ITPL in *T. castaneum* led to significant reduction in egg numbers due to failure in ovarian maturation and reduced survival of offspring after dsRNA injections at the pupal stage [45]. In *N. lugens*, ITPL was identified as a SFP transferred to females after mating. The function of ITPL as an SFP is a topic worth studying.

## Conclusion

From the proteomic analysis, we identified 94 putative secreted SFPs of *N. lugens* and the expression level of these proteins in the MAG was yielded by the DGE database. We found that proteins with more reliable evidence predicted to be secreted showed much higher expression in the MAG than other proteins, lending credibility to the detected SFPs. Comparative analyses revealed duplication and expansion of SFPs in *N. lugens* and the identification of novel SFPs in this species. Our results provide a foundation for future studies to investigate the functions of SFPs in *N. lugens* and are an important addition to the available data for comparative studies of SFPs in insects.

## Additional files

Additional file 1: Table S1. Primers used in this artical. This file lists the sequences of the primers used in the RT-qPCR analysis, as described in Methods. (XLSX 11 kb) Additional file 2: Table S2. Detailed annotation of detected proteins. This file gives the detailed annotation of detected proteins including the high confident SFPs and unconfirmed SFPs. The annotation process was described in Methods. (XLSX 134 kb) Additional file 3: Table S3. Statistics of function groups. This file gives a statistics analysis of the detected proteins of each function groups, as described in Methods. (XLSX 12 kb) Additional file 4: Table S8. Proteins only detected in MAG samples possessing a signal peptide. We analyzed the signal peptide of the proteins that were only detected from MAG samples. We identified 14 proteins with signal peptides, and their information, including their sequences, is provided in this file. (XLSX 31 kb) Additional file 5: Figure S1. Analysis of the expression profiles of seminal fluid protein genes in MRT dissections, MRT, and FRT by qRT-PCR. This file gives the RT-qPCR results of the detected proteins. (PDF 613 kb) Additional file 6: Table S4. Sequences of detected proteins. This file gives the nucleotide sequences and amino acid sequences of the detected proteins including the high confident SFPs and unconfirmed SFPs. (XLSX 236 kb) Additional file 7: Table S5. Comparison of SFPs between different insect species. This file gives the comparison results of SFPs between different insect species, as described in Methods. (XLSX 39 kb) Additional file 8: Table S6. Proteome data of each samples. This file gives the proteome raw data of each proteins samples. (XLS 1675 kb) Additional file 9: Table S7. Sequences of insect SFPs. This file gives the SFP sequences of other insect species used in the comparison with *N. lugens* SFPs. (XLSX 502 kb)

## Abbreviations

BPH: Brown planthopper
CB: Copulatory bursa
CDS: Coding sequence
DGE: Digital gene expression
EGF: Epidermal growth factor
FRT: Female reproductive tract
ITPL: Ion transport peptide like
MADF: mesencephalic astrocyte-derived neurotrophic factor
MAG: Male accessory gland
MRT: Male reproductive tract
RNAi: RNA interference
RPKM: Reads Per Kilo bases per Million mapped Reads
RT-qPCR: Reverse-transcription quantitative PCR
SFPs: Seminal fluid proteins
SR: Seminal receptacle
TE: Testes
VD: vas deferen
