# Supplementary material for: Seminal fluid protein genes of the brown planthopper, Nilaparvata lugens
Source: BMC Genomics. 2016 Aug 18;17:654. doi: 10.1186/s12864-016-3013-7 (PMC4990865; doi:10.1186/s12864-016-3013-7)
Supplement: Additional file 5: Figure S1. — Analysis of the expression profiles of seminal fluid protein genes in MRT dissections, MRT, and FRT by qRT-PCR. This file gives the RT-qPCR results of the detected proteins. (PDF 613 kb) [file 12864_2016_3013_MOESM5_ESM.pdf]

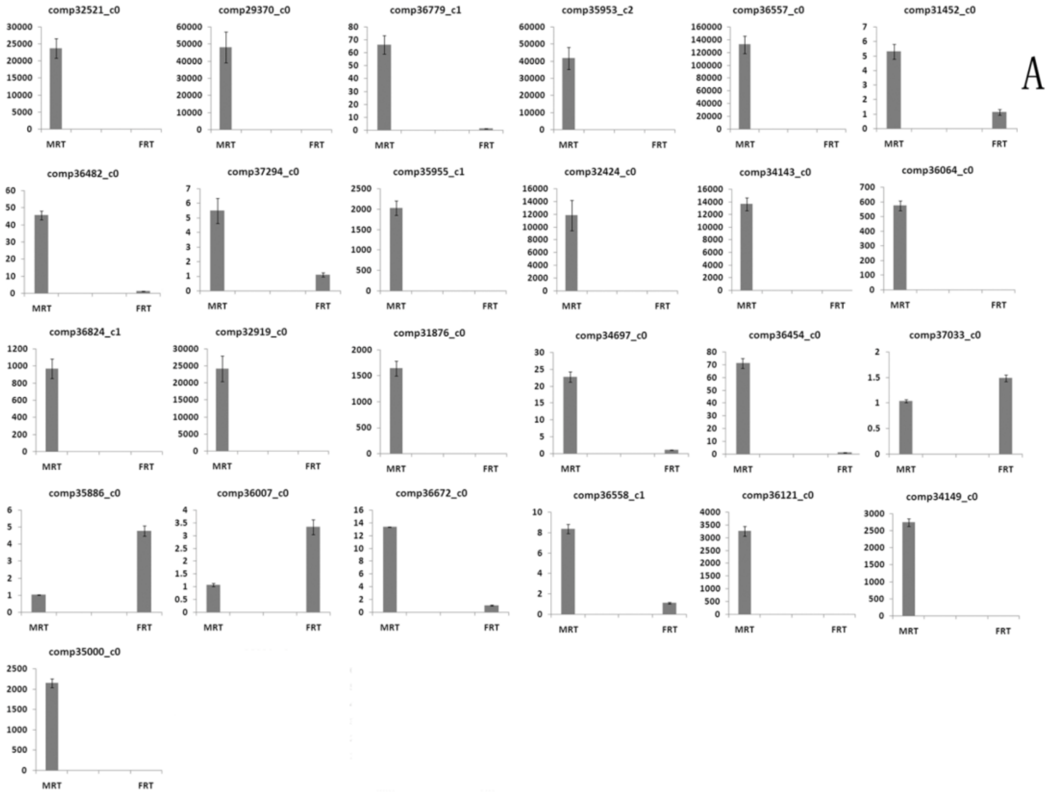
A
comp32521_c0
comp29370_c0
comp36779_c1
comp35953_c2
comp36557_c0
comp31452_c0
comp36482_c0
comp37294_c0
comp35955_c1
comp32424_c0
comp34143_c0
comp36064_c0
comp36824_c1
comp32919_c0
comp31876_c0
comp34697_c0
comp36454_c0
comp37033_c0
comp35886_c0
comp36007_c0
comp36672_c0
comp36558_c1
comp36121_c0
comp34149_c0
comp35000_c0
MRT
FRT

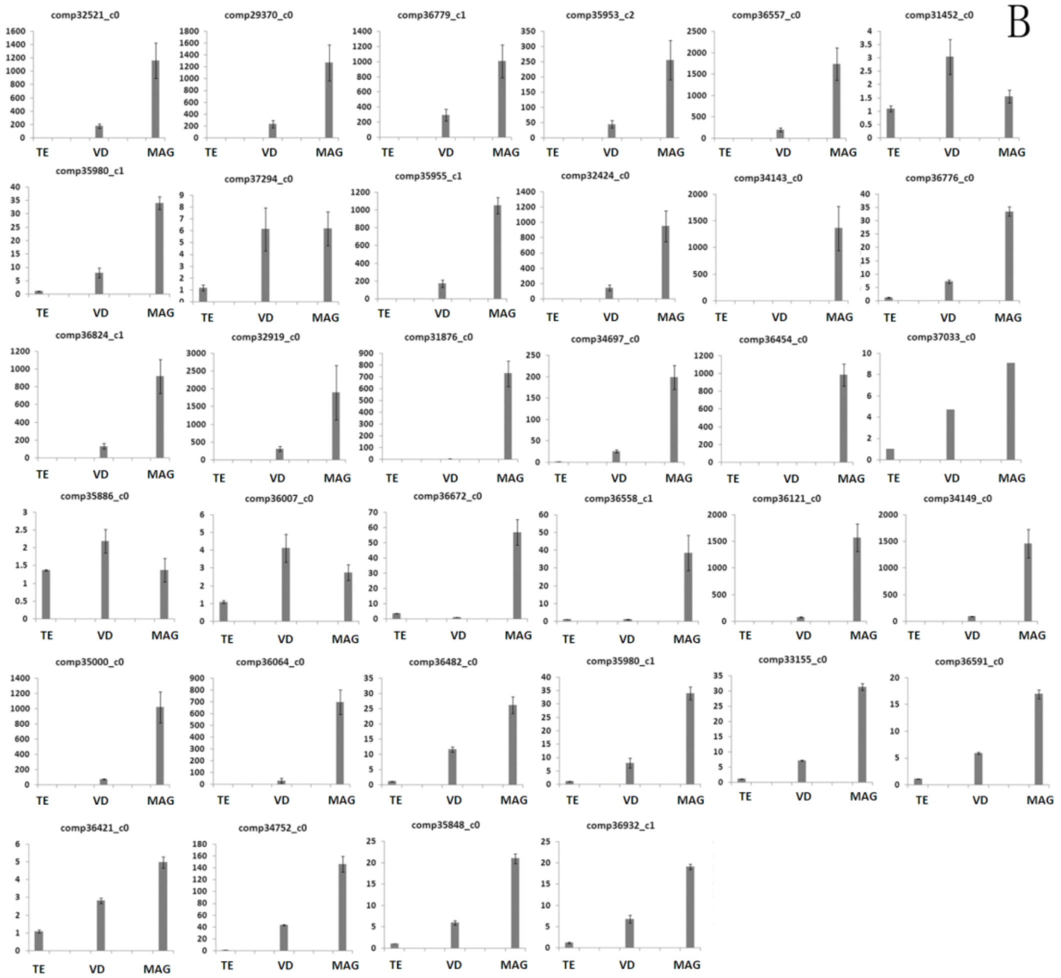
B
comp32521_c0
comp29370_c0
comp36779_c1
comp35953_c2
comp36557_c0
comp31452_c0
comp35980_c1
comp37294_c0
comp35955_c1
comp32424_c0
comp34143_c0
comp36776_c0
comp36824_c1
comp32919_c0
comp31876_c0
comp34697_c0
comp36454_c0
comp37033_c0
comp35886_c0
comp36007_c0
comp36672_c0
comp36558_c1
comp36121_c0
comp34149_c0
comp35000_c0
comp36064_c0
comp36482_c0
comp35980_c1
comp33155_c0
comp36591_c0
comp36421_c0
comp34752_c0
comp35848_c0
comp36932_c1
TE
VD
MAG

Supplemental Figure: Analysis of the expression profiles of seminal fluid protein genes in MRT dissections, MRT, and FRT by qRT-PCR. Total RNA was extracted from MRT, FRT and MRT dissections, including testes (TE), vas deferens (SD) and male accessory glands (MAG) individually. Samples were used for the analysis of expression of seminal fluid protein genes using qRT-PCR. The relative expression levels of each gene in each tissue were normalized using the N. lugens 18 s rRNA Ct values. The ΔΔCt method was used to measure relative transcript levels in each tissue. A: qRT-PCR results for MRT and FRT. B: qRT-PCR results for MRT dissections.
